# Water and Health

**Published:** 1891-11

**Authors:** 


### WATER AND HEALTH.

Water, says Dr. Lauder Brunton, is the most universal solvent in the world. It is not only useful to wash out our closets and flush our drains, it has a similar effect in our bodies, and tends to wash away the waste products from the cells of which our organs are composed, to clear out the uric acid, urea, and phosphates through our kidneys, and thus prevent renal or vesical calculi, and also to wash out our liver and prevent gall-stones, while it helps to keep the bowels in action. The liver especially is an organ which suffers from want of water, and I never see a gall-stone without asking the patient, " How much water do you drink ?" Almost invariably the answer is, "I hardly ever touch water. I am not a thirsty person ; " and on one occasion a lady called for a particular teacup, which held little more than a thimbleful, in order to show me how much she drank. On reckoning how much water she took in the twenty-four hours, it came as nearly as I could calculate, to sixteen fluid ounces. What wonder, then, that she had a gall-stone! The poor liver had not a chance to make decent fluid bile, and naturally there was a deposit. By making such people drink a big tumbler of water, and especially hot water, every morning, with or without some Carlsbad salts added to it, and, if necessary, repeating the hot water once or twice more in the day, the renewed formation of gall-stones may frequently be averted, and symptoms of biliary colic, to say nothing of so-called "biliousness," may be prevented for many years, or perhaps entirely. The (Carlsbad) process may be carried on at home by means of hot water either alone or with the addition of a small quantity of some saline, such as bicarbonate or nitrate of potash. In cases where the patient dislikes hot water alone, a slice of lemon thrown on the top of it gives it a slightly agreeable taste, and may overcome the patient's repugnance.

